# Cam morphology and inguinal pathologies: is there a possible connection?

**DOI:** 10.1007/s10195-017-0470-y

**Published:** 2017-09-18

**Authors:** G. N. Bisciotti, F. Di Marzo, A. Auci, F. Parra, G. Cassaghi, A. Corsini, M. Petrera, P. Volpi, Z. Vuckovic, M. Panascì, R. Zini

**Affiliations:** 1 Qatar Orthopaedic and Sport Medicine Hospital, FIFA Center of Excellence, Doha, Qatar; 2 Ospedale Unico della Versilia, Asl Nordovest, Lido di Camaiore, Lucca, Italy; 3 UOS angiografia e radiologia interventistica, Ospedale delle Apuane, Massa-Carrara, Italy; 4 Centro Studi Kinemove Rehabilitation Centers, Pontremoli and La Spezia, Italy; 5 FC Internazionale Medical Staff, Milan, Italy; 6 University of Ottawa, Ottawa, Canada; 7 Department of Knee Orthopaedic and Sport and Traumatology Unit, Humanitas Research Hospital, Rozzano, Italy; 8 Ospedale San Carlo di Nancy—GVM Care and Research, Rome, Italy; 9 Maria Cecilia Hospital—GVM Care and Research, Cotignola, Italy

**Keywords:** FAI syndrome, Cam morphology, Pincer morphology, Groin pain syndrome

## Abstract

**Background:**

To analyse the prevalences of the cam and pincer morphologies in a cohort of patients with groin pain syndrome caused by inguinal pathologies.

**Materials and methods:**

Forty-four patients (40 men and 4 women) who suffered from groin pain syndrome were enrolled in the study. All the patients were radiographically and clinically evaluated following a standardised protocol established by the First Groin Pain Syndrome Italian Consensus Conference on Terminology, Clinical Evaluation and Imaging Assessment in Groin Pain in Athlete. Subsequently, all of the subjects underwent a laparoscopic repair of the posterior inguinal wall.

**Results:**

The study demonstrated an association between the cam morphology and inguinal pathologies in 88.6% of the cases (39 subjects). This relationship may be explained by noting that the cam morphology leads to biomechanical stress at the posterior inguinal wall level.

**Conclusions:**

Athletic subjects who present the cam morphology may be considered a population at risk of developing inguinal pathologies.

**Level of evidence:**

Level IV, Observational cross-sectional study.

## Introduction

**Fig. 1:**
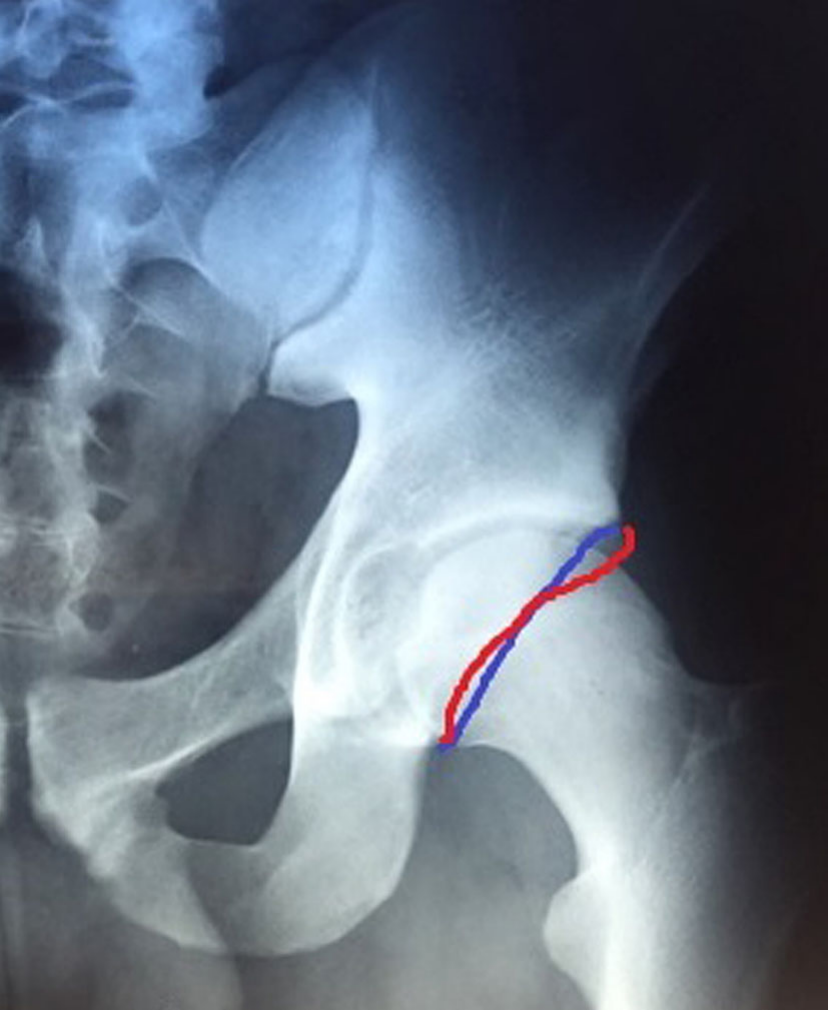
The crossover sign (COS) in a patient with the pincer morphology (overlap between the anterior and the posterior walls of the acetabulum). In a hip with normal anteversion, the line of the anterior wall lies medial to the line of the posterior wall, while the line of the posterior wall crosses the line of the anterior wall in acetabular retroversion

**Fig. 2:**
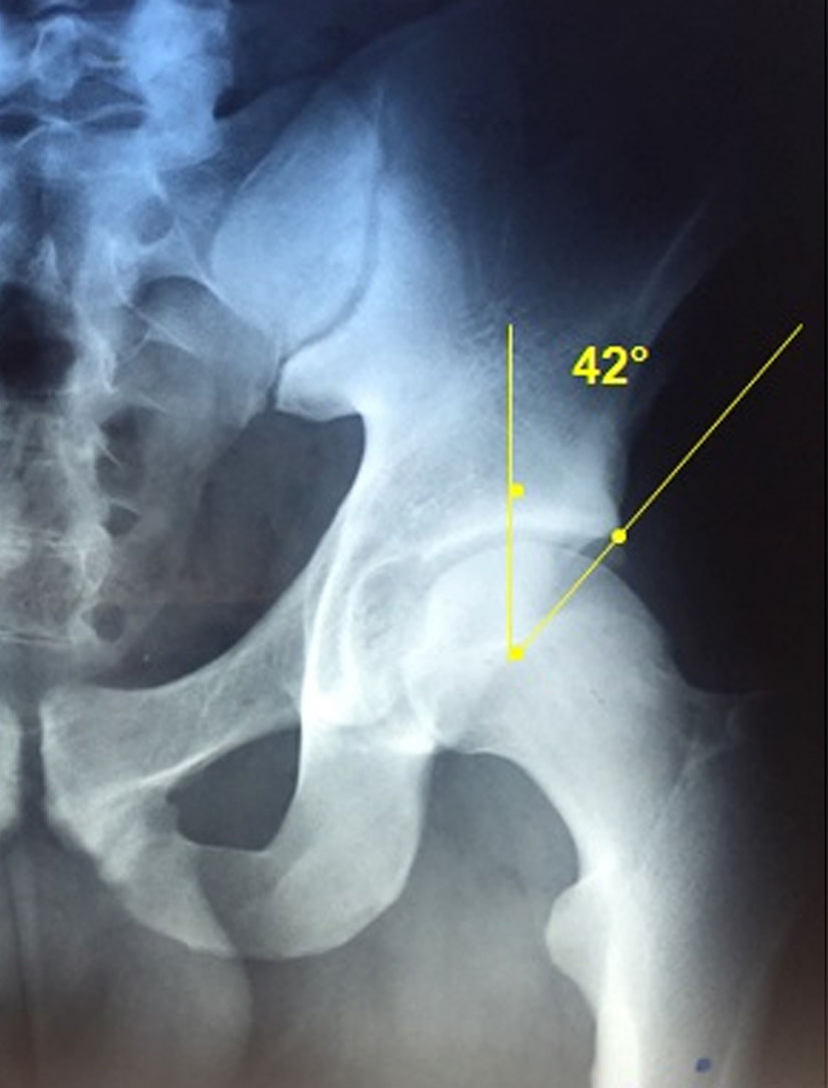
Centre-edge angle (CEA) measurement on an AP pelvis X-ray. The CEA is the angle between the line connecting the centre of the femoral head and the acetabulum and the line perpendicular to the line connecting the sit bones of the ischial tuberosity. A value of ≥40° is consistent with a pincer morphology

Femoroacetabular impingement (FAI) is a condition characterised by the presence of abnormal contact between the articular rim of the acetabulum and the proximal femur at the level of the femoral head-neck junction [1, 2]. FAI was first described by Smith-Petersen in the 1930s [3–5]. It was subsequently described in a more specific and detailed manner by Ganz in 1991 [6]. FAI presents in two main forms: the pincer and cam morphologies. The pincer morphology is characterised by over-coverage of the femoral head by the acetabulum, which leads to abnormal contact between the femoral neck and acetabular rim [1, 2, 7]. On the other hand, the cam morphology refers to an abnormal configuration of the femoral head in which an osteochondral bump is present at the femoral head-neck junction, leading to a decrease in the normal femoral head-neck offset [1, 2, 7]. Also, the pincer and cam morphologies may be present simultaneously, leading to a “mixed” type of FAI [8–10]. In the pincer morphology, the excessive acetabular coverage is detected from the presence of the crossover sign (COS) or the posterior wall sign in an anteroposterior (AP) pelvis X-ray [11, 12], as shown in Fig. 1. The COS is often a sign of acetabular retroversion [11, 12], but it is important to note that COS can sometimes also be present in the absence of acetabular retroversion [13]. Different morphologies and sizes of the anterior inferior iliac spine may create a COS image, even in the absence of true acetabular retroversion [14]. Acetabular coverage may also be assessed by measuring the lateral centre-edge angle (CEA). The CEA is the angle between the line connecting the lateral edge of the acetabulum with the centre of the femoral head and the line perpendicular to the line connecting the sit bones of the ischial tuberosity. A CEA measuring ≥40° is consistent with a pincer morphology [7]. Measurement of the CEA is shown in Fig. 2. The radiographic evaluation of a cam morphology is performed in the Dunn view taken with the hip flexed 45° or 90° and abducted 20°, and with the X-ray beam directed in the AP plane [11].

**Fig. 3:**
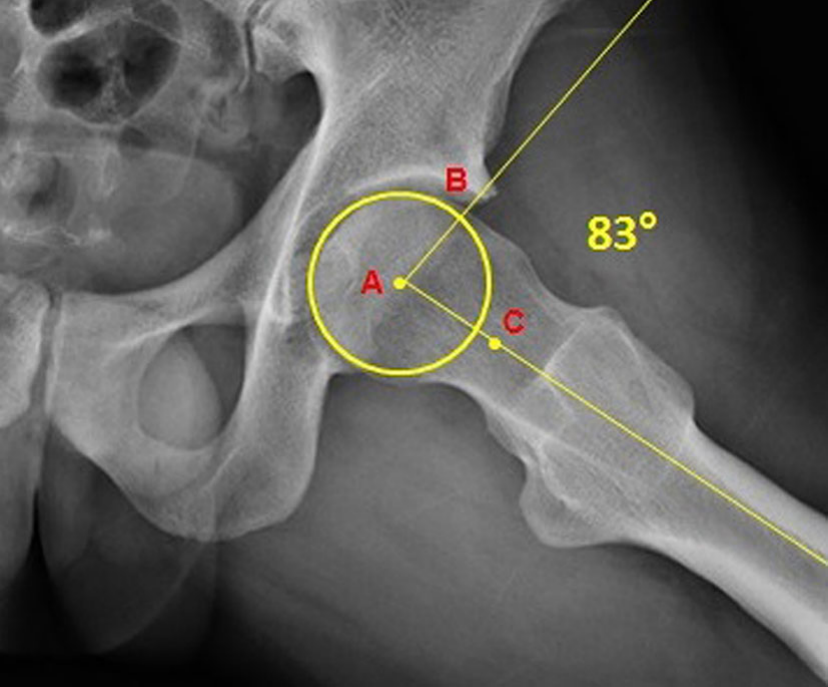
Alpha angle (α) measurement on a Dunn view X-ray. α is defined by drawing the best-fit circle around the femoral head and identifying the point at which the femoral head profile leaves the circle. A line is drawn between the centre of this circle (**a**) and the identified point (**b**). A second line is drawn between point A and the axis of the femoral neck, which is defined by connecting the centre of the femoral head with the centre of the femoral neck (**c**). The angle between these two lines is the α. An α ≥55° is considered to provide radiographic evidence of a cam morphology

Cam morphology impingement is then identified by measuring the alpha angle (α) on the Dunn view X-ray. Alpha is measured by drawing the best-fit circle around the femoral head and identifying the point at which the femoral head protrudes from the best-fit circle. Alpha is the angle between the line from this point to the centre of the best-fit circle and the axis of the femoral neck (Fig. 3). An α measuring ≥55° is considered to represent radiographic evidence of a cam morphology [15, 16]. However, it is important to highlight that FAI syndrome can be diagnosed only in the presence of a triad comprising symptoms, clinical signs and radiographic findings [17]. FAI, especially the cam morphology, may affect the anterosuperior chondrolabral junction and induce chondrolabral separation and acetabular chondral delamination [17]. The concept of “inguinal pathologies”, in accord with the Groin Pain Syndrome Italian Consensus Conference on Terminology, Clinical Evaluation and Imaging Assessment in Groin Pain in Athlete [18], refers to a spectrum of conditions that include inguinal hernia, posterior inguinal wall weakness, attenuation of the conjoined tendon, injury at the insertion of the rectus abdominis muscle, partial avulsion/tearing of internal oblique muscle fibres from the insertion of the symphysis, and tearing of the internal and/or external oblique muscle(s) at the aponeurosis level [18]. These inguinal pathologies are an important cause of groin pain syndrome (GPS) in athletes [18]. Recent studies have suggested that FAI (especially the cam morphology) and GPS are frequently presented simultaneously [7, 19, 20]. In fact, although the cam morphology and inguinal pathologies were historically considered to be distinct entities, we now reinforce the concept of the anatomical continuity of the pubic symphysis [19, 20]. According to this concept, an excessive functional load (especially torsional) at the level of the symphysis causes both of these clinical conditions simultaneously [7, 18–20]. This functional overload can cause progressive deformation of the femoral head, leading to both a cam morphology and weakness of the posterior inguinal wall, thus weakening neighbouring anatomical structures (i.e. the external and internal oblique muscle aponeuroses, the transverse abdominis, the conjoint tendon and the inguinal ligament). Since this has important clinical implications—especially for the type of treatment (either clinical or surgical) to be performed—there is a need to perform studies that can validate this postulated association of the cam morphology with inguinal pathologies. These studies should also allow us to propose some hypotheses explaining the common etiopathogenesis of these two conditions, as the mechanism that leads from a cam morphology to secondary breakdown of inguinal anatomical structures is unclear [21].

The aim of the cross-sectional study reported in the present paper was to analyse the prevalences of the cam and pincer morphologies in a cohort of patients with GPS. Based on the results, we propose a mechanical mechanism for the relationship between the cam morphology and inguinal pathologies, based on the biomechanical influence exerted by the change in the range of motion of the hip joint caused by the cam morphology at the posterior inguinal wall level.

## Materials and methods

### Study design

We performed an observational cross-sectional study of 44 patients suffering from GPS, each of whom underwent a laparoscopic repair of the abdominal wall. Each patient met the eligibility criteria for inclusion in the study. Ethical committee approval and written consent from each participant were obtained. The STROBE statement [22] supplemented by the TIDieR checklist and guide [23] were consulted for the study design, as were both the Minimal Reporting Standard for Groin Pain in Athlete [24] and the Groin Pain Syndrome Italian Consensus Conference on Terminology, Clinical Evaluation and Imaging Assessment in Groin Pain in Athlete (GPSICC) [18].

### Setting

All patients who presented at our Groin Pain Clinical Centre complaining of GPS during the period between December 2015 and January 2017 were initially considered (85 subjects).

All patients were examined by an experienced sports medicine physician, an abdominal surgeon and two radiologists in a blinded manner. For the clinical assessment, the guidelines indicated in the GPSICC were used [18].

### Eligibility criteria

The inclusion criteria adopted—in accordance with the guidelines established by the GPSICC—ensured that we only included patients who presented symptoms of the GPS clinical framework [18]. The exclusion criteria—again in accordance with the guidelines established by the GPSICC—made sure that the study excluded all patients who showed acute symptoms of the GPS clinical framework due to indirect muscle injury of the adductors, iliopsoas and rectus abdominis muscles. Furthermore, all of the patients who showed GPS clinical framework symptoms caused by a pubic symphysis stress fracture were excluded, as were those with apophysitis at the levels of the anterior inferior and superior iliac spine.

### Participants

In accordance with the abovementioned eligibility criteria for the study, 31 subjects who showed indirect muscle injury at the level of the adductors, ileopsoas and rectus abdominis muscles, 2 subjects with a pubic symphysis stress fracture, 5 subjects with apophysitis at the anterior inferior iliac spine, and 3 subjects with apophysitis at the anterior superior iliac spine level were excluded. Applying the abovementioned exclusion criteria, 44 patients were enrolled in the study. Subsequently, all of the subjects underwent a TAPP [25] laparoscopic repair of the posterior inguinal wall performed by the same surgeon.

### Demographic data

The following demographic data were recorded: gender, age, height, body weight, nationality, the subjectively judged rapidity of onset of symptoms (i.e. gradual or acute onset), the duration and laterality of the symptoms, the types of treatments that the subjects underwent previously, and the types and levels of sporting activities that they engaged in. Forty men and 4 women for whom the mean age, height and body weight were respectively 25.86 ± 3.21 years, 177.51 ± 5.72 cm and 75.25 ± 9.21 kg were enrolled in the study. Nationality was determined by passport or identity card, and all the subjects came from Italy. All the subjects experienced a gradual onset of symptoms. The average duration of GPS symptoms was 14.21 ± 3.3 months. Twenty-four subjects (54.5%) complained of bilateral pain and 20 of unilateral pain (45.5%). All subjects underwent and failed a trial of conservative management (i.e. specific physiotherapeutic treatments, strengthening and stretching) lasting 6.31 ± 5.83 months on average. All of the subjects practised sports (20 at the professional level and 24 at the amateur level). The types of sporting activities performed by the subjects, the levels of activity and the years of practice are shown in Table 1.

**Table 1:** Types of sporting activities performed by the subjects of the study, the levels of activity and the years of practice

Sporting activity	Professionals	Amateurs	Years of practice (average ± standard deviation)
Soccer 30 (68.1%)	17 (56.7%)	13 (43.3%)	19.6 ± 4.7
Basketball 1 (2.3%)	–	1 (100%)	12
Volleyball 3 (6.9%)	1 (33.3%)	2 (66.7)	12.2 ± 4.8
Ski 1 (2.3%)	1 (100%)		11
Tennis 1 (2.3%)		1 (100%)	7
Dance 1 (2.3%)	1 (100%)	–	10
Other recreational activities 7 (15.8%)	–	7 (100%)	9.8 ± 4.3

### HAGOS questionnaire

Before the clinical examination, the patients were asked to fill out the HAGOS (Copenhagen Hip and Groin Outcome Score) questionnaire. The HAGOS questionnaire is a patient-reported outcome measure to assess pain, physical activity and quality of life in patients with hip and/or groin pain [26]. In this study, the form of the HAGOS questionnaire that has been validated for Italians was used [27].

### Physical examination

All patients were examined by an experienced sports medicine physician and an abdominal surgeon in a blinded manner. A standardised protocol established by the GPSICC [18] was used in the clinical examination. This protocol includes the following assessments:

- Squeeze test (ST)
- Flexion abduction external rotation test (FABER)
- Flexion adduction internal rotation test (FADIR)
- Hip joint internal rotation measurement from a sitting position with leg bent to 90° (hip IR)
- Hip joint external rotation measurement from a sitting position with leg bent to 90° (hip ER)
- Inguinal canal assessment (ICA)

During the ST, FABER and FADIR tests, the patient was asked to quantify the pain according to a VAS scale [28].

The hip IR and hip ER values recorded during the physical examination were compared with the normal hip IR and hip ER values reported in the literature: 41° and 45°, respectively [29, 30].

### Radiographical evaluation

The radiographical evaluation was conducted by two radiologists according to the protocol established by the GPSICC [18], which includes

1. *X-ray examination*.
  Radiography routinely includes the following exams:
  Anteroposterior view in the upright position (AP1)
  Anteroposterior view in the upright position and alternately on each foot (flamingo view) (AP2)
  Dunn view (D).
    The following information was obtained from the radiographic assessment:
    Presence or absence of the crossover sign (COS) from AP1
    Enlargement and/or erosion and/or sclerosis of the symphysis (SS) from AP1
    Calculation of CEA from AP1
    Symphysis asymmetry (SA) from AP2
    Calculation of the alpha angle (α) from D
    The α measurements were performed from the 12 o’clock to the 3 o’clock positions on the best-fit circle (Fig. 4). Measurements of the CEA angle and the α were performed using the angle-measurement and circle-drawing tools of the Kinovea 08.15 software package (Hip2Norm; Software Informer Technologies, Inc.). Two different radiologists performed the measurements in a blinded fashion.

**Fig. 4:**
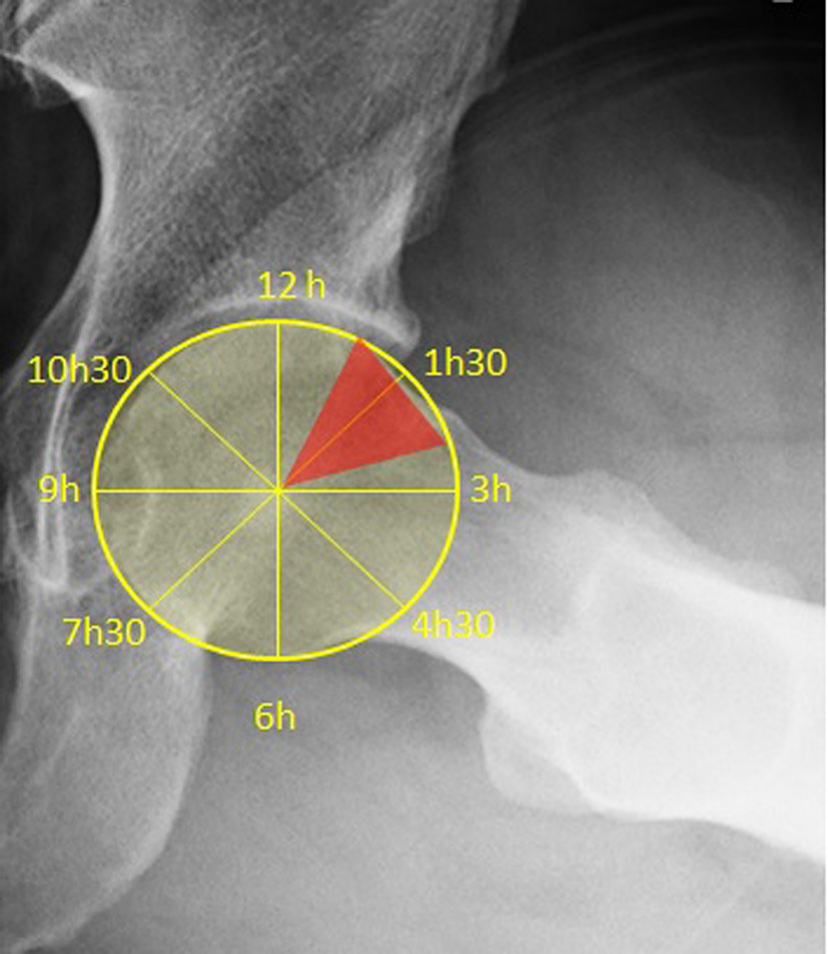
Measurement of α performed throughout the cranial hemisphere from 12 o’clock to 3 o’clock. The typical location of pathological α was between 1 and 2.30 o’clock
2. *Ultrasound (US) examination*.
  The US examination, performed by a radiologist with expertise in the evaluation of inguinal pathologies, was done according to the protocol established by the GPSICC [18]. The sports medicine physician responsible for the physical examination and an abdominal surgeon were also present during the US evaluation, in agreement with the guidelines of the GPSICC [18]. The US examination involved
  Assessment of the muscle–tendon unit of the abdominal and adductor muscles (AMTU)
  Dynamic assessment of the inguinal canal structures (DAIC)
  Assessment of internal organs (AIO)
  Assessment of the urinary tract and external genitalia (AUT)

The inguinal pathologies, diagnosed based on clinical and US examinations, were classified according to the European Hernia Society classification [31] as a direct hernia (M1, M2, M3) or an indirect hernia (L1, L2, L3). Cases where the US examination did not show a true hernia but a bulge in the posterior inguinal wall were classified (according to the GPSICC [18]) as posterior inguinal wall weakness (PIWW).

### Surgical repair of the abdominal wall

All of the subjects underwent a laparoscopic repair of the abdominal wall, consisting of mesh positioning with the TAPP technique [25]. All of the laparoscopic repairs were performed by the same surgeon.

### Statistical analysis

The basic statistical indices (average ± standard deviation) were calculated for each variable. The data distribution was assessed with the Kolmogorov–Smirnov test. Since the data distribution was nonparametric, the correlation between the considered parameters was calculated with Spearman’s rank correlation coefficient. The statistical significance of a difference between recorded values was calculated with the Wilcoxon test.

The *k* values for interobserver reliability for the COS, SS, CEA and α measurements were also calculated.

The significance of the difference between a recorded value and the corresponding expected value was calculated with the chi-squared test. Statistical significance was set at *p* < 0.05.

## Results

### HAGOS questionnaire

The average HAGOS value was 42.41 ± 14–50 (range 24–68).

### Physical examination

The average hip ER value in the studied population was 44.29 ± 2.54° (range 35–50°). The average hip ER value in hips with α < 55° was 44.90 ± 3.1° (range 40–50°), while it was 44.45 ± 3.78 (range 41–50°) in hips with α > 55°. This difference was not statistically significant. The average hip IR value in the studied population was 38.27 ± 4.20° (30–50°). The average hip IR value in hips with α < 55° was 40.69 ± 3.11, while the average hip IR value in hips with α > 55° was 38.44 ± 4.20. This difference was statistically significant (*p* < 0.05). The VAS score for ST in hips with α < 55° was 2.81 ± 1.92 (range 0–6), while the VAS score for ST in hips with α > 55° was 4.78 ± 2.22 (range 1–9). This difference was statistically significant (*p* < 0.005). The VAS score for FABER in hips with α < 55° was 1.64 ± 1.48 (range 0–5), while the VAS score for FABER in hips with α > 55° was 5.08 ± 2.33 (range 0–9). This difference was statistically significant (*p* < 0.005). The VAS score for FADIR in hips with α < 55° was 1.47 ± 1.32 (range 0–5), while the VAS score for FADIR in hips with α > 55° was 5.17 ± 2.20 (range 2–9). This difference was statistically significant (*p* < 0.005).

### Radiographic evaluation

The mean α value was 64.87 ± 11.53° (range 42–80°) and 65.97 ± 11.12° (range 50–80°) for the right hip and left hip, respectively. The recorded α value was statistically different from the reference value (i.e. 55°; *p* < 0.05). The cam morphology was bilateral in 33 subjects (75%). An α > 55° was associated with inguinal pathologies in 88.6% of the cases (39 subjects). The inguinal pathology was bilateral in 23 subjects (52.3%). The inguinal pathologies observed and their associations are presented in Table 2. SS was present in 45.45% of the cases (20 subjects). The average CEA value was 38.52 ± 2.38° (range 35–42°). The percentage with CEA values of >40° was 13.63% (6 subjects), while the percentage with the COS was 13.63% (6 subjects). The simultaneous presence of the COS, a CEA of >40°, and the cam morphology was observed in 13.63% of the cases (6 subjects). The average SA value was 3.52 ± 0.48 mm (range 2–4 mm). SA was present in 38.63% of the cases (17 subjects). The α was bilaterally >55° in 70.4% of the cases (31 subjects). The *k* values for interobserver reliability for COS, SS, CEA and α are shown in Table 3.

**Table 2:** The various inguinal pathologies observed and their associations

Inguinal pathology	Percentage of patients	Number of patients	Notes
Bilateral PIWW	29.5	13	
Right PIWW	27.2	12	
Left PIWW	11.3	5	
Unilateral M1	2.2	1	
Bilateral M1	13.6	6	
Unilateral M2	–	–	
Bilateral M2	4.4	2	
M1 + M2	4.4	2	
L1	6.8	3	1 Coupled to bilateral M2 and 2 coupled to bilateral M1

**Table 3:** Interobserver reliability for the presence of the crossover sign (COS), enlargement and/or erosion and/or sclerosis of the symphysis (SS), the centre-edge angle (CEA) and the alpha angle (α)

Structural feature	Interobserver reliability (*k* value)	Confidence interval (CI 95%)
COS	0.60	0.52–0.68
SS	0.73	0.68–0.79
CEA	0.77	0.70–0.84
α	0.76	0.69–0.83

### Ultrasound examination

The AMTU revealed

- Adductor tendinopathy (AT) in 34.09% of the cases (15 subjects); this was bilateral in 9.0% of the cases (4 subjects)
- Rectus abdominis tendinopathy (RT) in 11.36% of the cases (5 subjects); this was bilateral in all cases
- Adductor–rectus abdominis tendinopathy (ART) was present in 6.81% of the cases (3 subjects); this was bilateral in all cases

AIO and AUT resulted negative in all the examined subjects.

The AIO and the AUT did not reveal any abnormalities in any of the subjects.

**Fig. 5:**
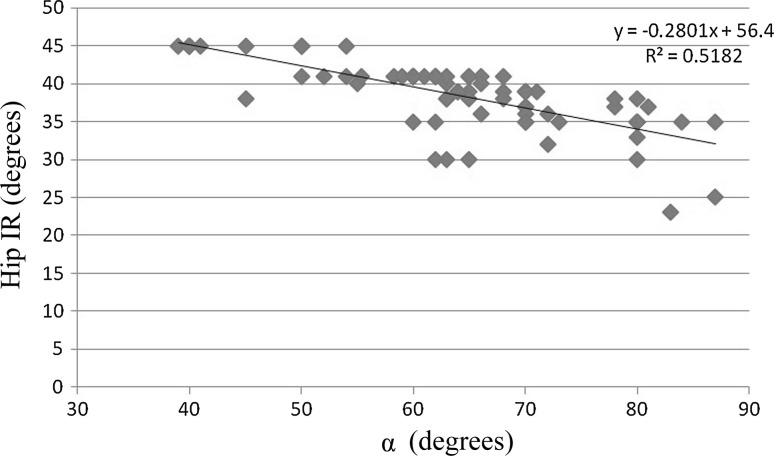
Correlation between the α value and the hip IR value

The hip internal rotation value found in this study was significantly correlated with the α value (*R* ^2^ = 0.52 *p* < 0.05). The correlation is shown in Fig. 5.

## Discussion

The findings of the present study show a strong association between the cam morphology and inguinal pathologies. In this observational cross-sectional study, inguinal pathologies and the cam deformity coexisted in 88.6% of the cases. The results of this study are in line with the results obtained in previous similar studies [21, 32, 33]. To our knowledge, among published studies focusing on the association between FAI and inguinal pathologies, this study includes the largest number of cases. The cam morphology is one of the most common deformities of the femur and is characterized by a nonspherical profile of the anterosuperior head-neck junction [34, 35]. This loss of spherical shape may cause a femoroacetabular impingement lesion and loss of hip internal rotation [36–38]. The aetiology of this deformity is not clear; some authors suggest that it is linked to a childhood disease such as subclinical Perthes disease [39–41] or to abnormal ossification of the proximal femur resulting from congenital factors [42]. However, it is interesting to note that some authors [40, 43] have reported a high percentage of the cam morphology in athletic populations, especially in those participating in soccer, handball and track and field. More recent studies [31, 32, 44] have confirmed these findings and suggest that participation in high-intensity sporting activities during adolescence is associated with an increased risk of the cam morphology. The cam morphology is common in subjects who engage in high-demand sporting activities (particularly soccer [44]), especially in adolescent populations who participate in these sporting activities before the age of 14 [45–47]. Soccer players have an increased risk of developing the cam morphology bilaterally and a more pronounced α than the general population [44]. This might be related to the fact that the cam morphology is, at least partly, considered a developmental deformity that may be triggered in adolescent subjects who partake in intensive sporting activity during the closure of the femoral head growth plate [31, 32, 44]. In the general asymptomatic population, the reported prevalence of the cam morphology is 14% [48, 49]. In this study, the recorded typical α dislocation was between 1 and 2:30 o’clock, with a mean value of 64.87 ± 11.53° for the right hip and 65.97 ± 11.12° for the left hip (range: 42–80° and 50–80°, respectively). An α of between 1 and 2:30 o’clock is typical of the most prominent cam morphology [31]. With regard to α, the* k* value for interobserver reliability (0.76; 95% CI 0.69–0.83) is in line with the results of previous studies [50, 51] and therefore demonstrates the validity of the measurement method adopted in this study. The weak association between the pincer and cam morphologies observed in this study can be explained by the fact that the majority of the subjects involved were male individuals (33 subjects), and it has been noted that the cam morphology is predominant in males [52]. Furthermore, in this study, the pincer morphology (i.e. the simultaneous presence of COS and CEA > 40°) was present in 6 subjects (13.63% of the cases), and those subjects also presented a cam morphology (i.e. α < 55°). For this reason, the clinical framework of those subjects can be definable as “mixed” FAI [8–10]. Therefore, in this study, we were not able to evaluate the effects of a “pure” pincer morphology at the inguinal canal level.

It is interesting to note that there is geographical variation in the cause of GPS. Some studies have shown that >50% of Australian athletes suffering from GPS had an incipient hernia [53], 58% of athletes in Denmark with GPS had adductor-related groin pain [54], 50% [55] and 56% [21] of athletes with GPS in the UK and Ireland, respectively, showed hip pathologies, while adductor-related groin pain was present in more than 60% of the cases in a population comprising 51% individuals from the Gulf Countries, 25% Africans, 7% Europeans and 14% from other countries [56]. In our studies, 51.7% (44/85) of the examined subjects showed inguinal pathologies. The differences between the data collected in our study and the data from other published studies may have several explanations. First, differences in the ethnic compositions of the populations studied may have influenced the observed pathologies [56]. Second, the populations in the relevant published studies had the following sporting-activity compositions: 51% Australian football, 14% athletics, 12% soccer and 23% other sports activities (in Australia) [53]; 66% football, 18% running and 16% other sports (in Denmark) [54]; 22% soccer, 21% rugby, 16% running and 41% other sports (in the UK) [55]; 37% soccer, 13% Gaelic sports, 16% running, 8% no regular sporting activity, 18% other sports and unknown sporting activity in 8% (in Ireland) [21]; 60% football, 7% futsal, 6% athletics, 5% handball and 22% other sports (mainly in the Gulf Countries and Africa) [56]; and 68% of the population observed in the present study played soccer while the remaining 32% participated in other sporting and recreational activities. This lack of homogeneity in sporting-activity composition among the populations considered in the various studies can, at least partially, explain the differences between the results obtained in those studies.

The differences between the results obtained in the various studies can also be explained to some degree by the fact that imaging techniques and the knowledge base for GPS have improved over time [56]. Finally, differences in the diagnostic approach and the definition of GPS used in the different studies should also be considered when attempting to explain the inhomogeneity in the results of the various studies. Some studies [56] used the Doha Consensus Classification of Terminology and Definitions of Groin Pain in Athlete [57], others used a different autonomous diagnostic approach [21, 53–55], while the present study employed the Groin Pain Syndrome Italian Consensus Conference on Terminology, Clinical Evaluation and Imaging Assessment in Groin Pain in Athlete [18].

In any case, is important to underline that—in agreement with our results—some other studies suggest that inguinal pathologies [53, 56] and hip pathologies [21, 55] are the entities that are most likely to cause GPS in those participating in kicking and change-of-direction sports, and that hip pathologies may be a major contributor to secondary pathologies of adjacent anatomical structures [56, 58].

FAI syndrome and inguinal pathologies were previously considered to be two separate clinical entities, but recent studies have suggested that both of these clinical conditions can exist simultaneously in athletes affected by GPS [7, 12, 20, 60]. It was recently shown that a cam deformity may be associated with GPS, especially in young high-level athletes [19, 32, 57, 59–62]. The strong association between the cam deformity and inguinal pathologies (88.6%) found in this study confirms findings from previous studies [21, 31, 32].

In agreement with the results of our study, several authors have suggested that hip pathologies are implicated in the onset of secondary pathologies in surrounding anatomical structures [21, 58, 63, 64]. The mechanism responsible could vary and may depend on the specific type of hip pathology involved, the sex and age of the subject, and the sporting activity [21]. In the specific case of the cam morphology, we propose a mechanical-type hypothesis to explain the association between the cam morphology and inguinal pathologies. Indeed, during hip internal rotation when the cam morphology is present, the proximal femur impinges with the acetabular rim, limiting the range of motion (ROM) [65]. Moreover, the limited hip internal rotation (38.27 ± 4.20° versus a reference value of 41°; *p* < 0.05 [27, 30, 66]) observed in this study was associated with an abnormally high α (*R* ^2^ = 0.52, *p* < 0.05); this finding is in accord with the findings of previous studies [33, 52, 53]. This type of biomechanical limitation in athletes may lead to a compensatory stress at the pubic symphysis level [19] and a higher load on the abdominal wall muscles, which may then induce a secondary injury at either the muscle–tendon complex anterior to the inguinal wall or at the posterior inguinal wall level [19, 67, 68].

The proposed mechanical explanation for the association between the cam deformity and inguinal pathologies is based on the concept of the anatomical continuity of the pubic symphysis [18, 68]. This concept considers the symphysis to be one part of a broader anatomical system consisting of four layers [19, 68]:

1. Layer I or the osteochondral layer, comprising the femur, pelvis and acetabulum
2. Layer II or the inert layer, which includes the labrum, joint capsule, ligamentous complex and ligamentum teres
3. Layer III or the dynamic layer, comprising all of the musculature around the hemipelvis, including the lumbosacral and pelvic floor and the posterior wall of the inguinal canal
4. Layer IV or the neural layer, including the thoracolumbosacral plexus.

These four layers are intimately interconnected, and a dysfunction at any level can impact on other levels [68].

In athletes with a cam morphology, the functional ROM required in athletic competition is often greater than the limited physiological range of motion [19, 68]. In previous studies, it was hypothesised that the reduction of ROM caused by the cam morphology may increase loading and mechanical stress at the pubic symphysis level and on surrounding structures [69]. It is not totally clear, however, which of the structures limit the hip ROM [69]. Some authors have suggested that the ROM restriction is caused by an inflammatory process that is a response to increased loading, which may lead to capsular tightness. Such a mechanism would be similar to what is observed in overhead athletes [70] in terms of a restriction in the shoulder ROM [71].

Therefore, the limitation on the ROM at layer I (particularly at the femoroacetabular level) can be compensated for by hypermobility of the symphysis pubic joint. Hypermobility of the symphysis may affect layer III, leading to increased stress on the posterior inguinal wall and favouring the onset of inguinal pathologies [18, 19, 68]. This hypothesis is supported by the fact that some laboratory-based studies have demonstrated that a reduced ROM at the hip joint level leads to overloading of the symphysis [72]. Furthermore, this hypothesis is in line with the findings of several studies in which GPS was found to occur during high-energy twisting activities where abnormal hip ROM and the resultant pelvic motion concentrates shearing stress across the pubic symphysis and posterior inguinal wall [73–75]. In our study, the influence of the cam morphology on the hypermobility of the symphyseal joint was confirmed by how often SS (45.45%, 20 subjects), SA (38.36%, 17 subjects) and AT (34.09%, 15 subjects) were observed in our series. SA > 2 mm and the presence of SS and AT are signs of symphysis instability [18].

Most theories consider overloading to be the aetiology for inguinal pathologies. Repetitive, sudden and forceful pelvic twisting movements may produce shearing forces at the pubic symphysis level, leading to avulsion or tearing of the muscles and ligaments anterior to the inguinal wall [76] (i.e. aponeuroses of the external and internal oblique muscles, transverse abdominis, conjoint tendon and inguinal ligament). Furthermore, the overuse syndrome resulting from this biomechanical stress may cause the disruption of the insertion of the rectus abdominis muscle at the symphysis level [68, 77]. Another theory postulates that biomechanical stress may cause a laxity in the posterior inguinal wall, leading to a bulge that compresses the genital branch of the genitofemoral, ilioinguinal and iliohypogastric nerves [78, 79].

It is important to note that, in this study, the cam morphology was bilateral in 33 subjects (75%) while the inguinal pathology was bilateral in 23 subjects (52.3%). This can be explained by the fact that the development of a symptomatic impingement which exerts stress on the inguinal canal requires a particular combination of different factors [1, 2, 7, 17] such as a certain type and level of activity, the existence of a dominant leg (e.g. the leg with which the subject kicks in soccer) and a pivot leg in the biomechanical model of the sport, and the presence of other anatomical abnormalities (e.g. lower limb asymmetry, functional alterations of the knee or ankle joint, spine-related problems, etc.).

Another important point is that when a surgeon is treating an athlete who has overlapping inguinal and hip pathologies, the surgeon must decide whether to treat only the inguinal or intra-articular hip pathology or to address both problems at the same time. This point has already been raised by several authors [19, 80]. Larson et al. [80] evaluated 37 subjects who were diagnosed with symptomatic GPS caused by inguinal pathologies and associated intra-articular hip pathologies (30 cases of FAI, 1 traumatic labral tear and 1 borderline dysplasia). The subjects were evaluated at a mean follow-up of 29 months (range 12–78 months) after surgery. The results showed that when surgery addressed either the inguinal pathology or the intra-articular hip pathology in isolation, the outcome was suboptimal. On the contrary, surgical management of both disorders concurrently or in a staged manner was found to lead to improved postoperative outcome scoring and an unrestricted return to sporting activity in 89% of the subjects.

Hammoud et al. [19] reported the results from a cohort of 38 professional athletes who underwent surgery for FAI syndrome (cam morphology), among whom 12 (32%) had previously undergone surgical intervention for inguinal pathologies. After additional surgical treatment for a cam morphology, all of the subjects were able to return to their sporting activities. Moreover, among the remaining 26 patients, 15 had symptoms of inguinal pathologies that subsequently resolved after surgical treatment. In this study, only 39% (15/39) of the athletes with both an inguinal pathology and a cam morphology showed complete resolution of the pain and returned to their sporting activities following cam morphology surgery alone.

It is therefore possible that there is a degree of cam morphology and a specific α value beyond which it is necessary to consider simultaneous surgery for intra-articular hip and inguinal pathology. Furthermore, it is also reasonable to suggest that, in the presence of a high degree of cam deformity, inguinal surgical procedures involving a mesh may lead to an unsatisfactory outcome after a long follow-up due to the persistence of mechanical stress. However, further studies are needed to confirm this hypothesis.

It is important to note that some authors recently proposed increasing the cut-off value of α to between 78° [81] and 83° [82].

Finally, another interesting finding of our study is that ST, FABER and FADIR tests and the HAGOS questionnaire [25, 26] demonstrated excellent sensitivities in the diagnosis of a cam morphology and inguinal pathologies. In particular, we underline the high sensitivity shown by the FADIR test in relation to an α > 55° (VAS score was 5.17 ± 2.20 when α > 55°; it was 1.47 ± 1.32 when α < 55°, *p* < 0.005). This results are in line with the results of other studies [17].

The most important limitation of this study was that all of the patients who were enrolled had presented for assessment a long time after the onset of symptoms (14.20 ± 3.2 months), so the clinical diagnosis for all patients was long-standing groin pain syndrome [18]. Further research should focus on investigating whether the same correlation between the cam morphology and inguinal pathologies can also be found in subjects with a shorter history of GPS. A second limitation of our study is the high percentage of soccer players included (68.1%). The high number of soccer players that enrolled could have influenced the average value of α. Further studies focusing on a different sports population would be helpful. Finally, an interesting development of this study would be the assessment of the long-term outcomes of inguinal surgery with a mesh in the presence of significant cam deformity.

In conclusion, this study showed a strong association between cam FAI and inguinal pathologies. This raises the question of whether a double intervention (i.e. hip arthroplasty and inguinal repair) is justified in cases of both associated groin pain syndrome and significant cam deformity. However, based on the nature of our study, it was not possible to demonstrate a cause–effect relationship between the cam morphology and inguinal pathologies, so future studies will be necessary to clarify the extent to which hip pathologies may induce secondary breakdown at surrounding anatomical structures.
